# Supplementary material for: High salt exacerbates acute kidney injury by disturbing the activation of CD5L/apoptosis inhibitor of macrophage (AIM) protein
Source: PLoS One. 2021 Nov 29;16(11):e0260449. doi: 10.1371/journal.pone.0260449 (PMC8629239; doi:10.1371/journal.pone.0260449)
Supplement: S1 Fig — (A) Serum from newly generated AIM-/- and AIM+/+ CD1 littermates were analyzed by immunoblotting in non-reducing condition using anti-AIM antibody (n = 3 each). No AIM protein was detected in AIM-/- serum. (B) Representative photos of immunohistochemistry for KIM-1 on day 3 after HS-IR in newly generated KIM-1-/- and the littermates KIM-1+/+ are shown (n = 3). Control (cont. without any treat) is also presented. No KIM-1 expression was induced after HS-IR in KIM-1-/- mice while KIM-1 was highly expressed in the corticomedullary junction area in KIM-1+/+ mice after HS-IR. (PDF) [file pone.0260449.s001.pdf]

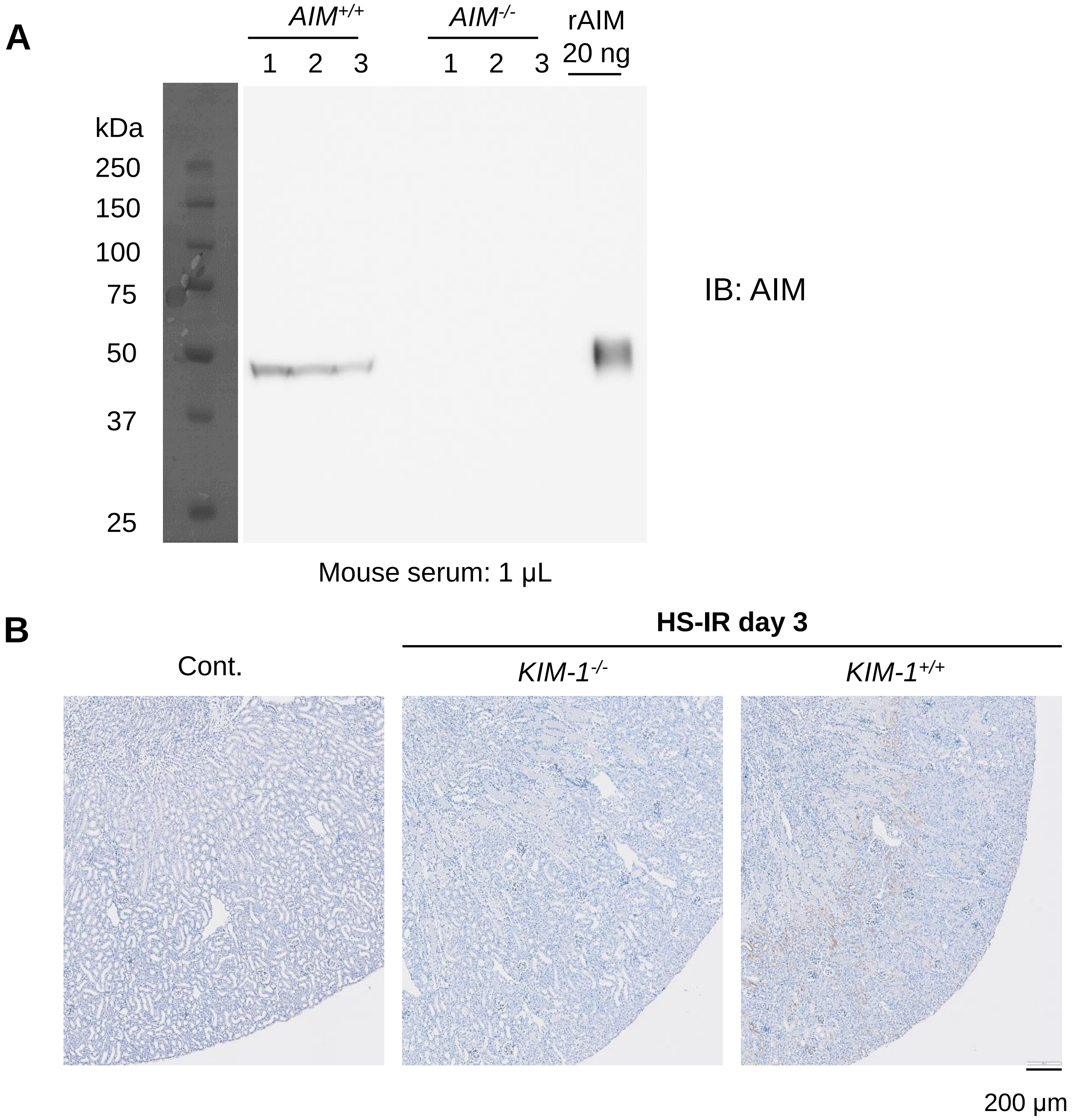

S1 Fig. The validation of the newly generated *AIM*$^{-/-}$ CD1 mice and the *KIM-1*$^{-/-}$ mice. **(A)** Serum from newly generated *AIM*$^{-/-}$ and *AIM*$^{+/+}$ CD1 littermates were analyzed by immunoblotting in non-reducing condition using anti-AIM antibody (*n* = 3 each). No AIM protein was detected in *AIM*$^{-/-}$ serum. **(B)** Representative photos of immunohistochemistry for KIM-1 on day 3 after HS-IR in newly generated *KIM-1*$^{-/-}$ and the littermates *KIM-1*$^{+/+}$ are shown (n = 3). Control (cont. without any treat) is also presented. No KIM-1 expression was induced after HS-IR in *KIM-1*$^{-/-}$ mice while KIM-1 was highly expressed in the corticomedullary junction area in *KIM-1*$^{+/+}$ mice after HS-IR.
