# Supplementary material for: High salt exacerbates acute kidney injury by disturbing the activation of CD5L/apoptosis inhibitor of macrophage (AIM) protein
Source: PLoS One. 2021 Nov 29;16(11):e0260449. doi: 10.1371/journal.pone.0260449 (PMC8629239; doi:10.1371/journal.pone.0260449)
Supplement: S2 Fig — (A) Percentage decrease in total renal blood flow after IR in IR (n = 8) and HS-IR (n = 9) mice. (B) Systolic blood pressure in IR (n = 5) and HS-IR (n = 6) mice on the indicated days after IR. No significant differences between HS-IR and IR were detected at any time point with Welch’s t-test. (PDF) [file pone.0260449.s002.pdf]

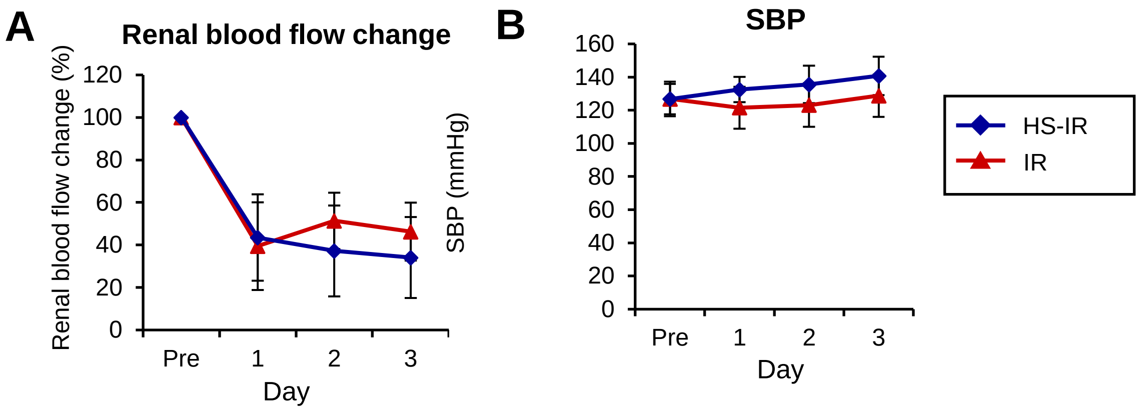

S2 Fig. Renal blood flow and systolic blood pressure changes after HS-IR.
(A) Percentage decrease in total renal blood flow after IR in IR (*n* = 8) and HS-IR (*n* = 9) mice. **(B)** Systolic blood pressure in IR (*n* = 5) and HS-IR (*n* = 6) mice on the indicated days after IR. No significant differences between HS-IR and IR were detected at any time point with Welch's t-test.
