# Supplementary material for: High salt exacerbates acute kidney injury by disturbing the activation of CD5L/apoptosis inhibitor of macrophage (AIM) protein
Source: PLoS One. 2021 Nov 29;16(11):e0260449. doi: 10.1371/journal.pone.0260449 (PMC8629239; doi:10.1371/journal.pone.0260449)
Supplement: S3 Fig — (A) F4/80-high or low cells in CD45+Ly-6G-CD11b+ cells prepared from kidneys (indicated in the upper panels) were analyzed for the intracellular expression of various pro-inflammatory factors in HS-IR and IR (n = 3 each) mice on day 3 after IR/sham. The results from the mice without any treatment (Cont.) are also presented (n = 3). Statistics: one-way ANOVA followed by Holm’s post hoc test. (B) QPCR analysis of the mRNA expression of fibrotic genes performed as in Fig 2H. (PDF) [file pone.0260449.s003.pdf]

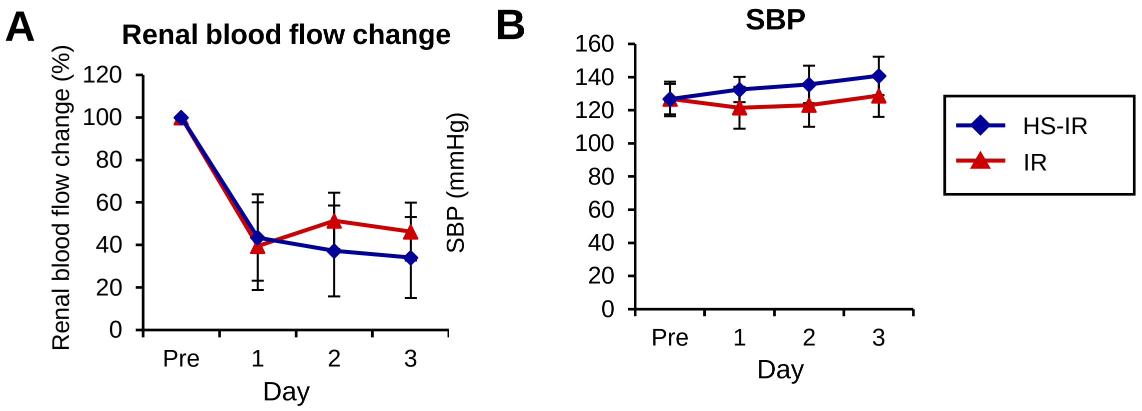

S2 Fig. Renal blood flow and systolic blood pressure changes after HS-IR.
(A) Percentage decrease in total renal blood flow after IR in IR (*n* = 8) and HS-IR (*n* = 9) mice. **(B)** Systolic blood pressure in IR (*n* = 5) and HS-IR (*n* = 6) mice on the indicated days after IR. No significant differences between HS-IR and IR were detected at any time point with Welch's t-test.

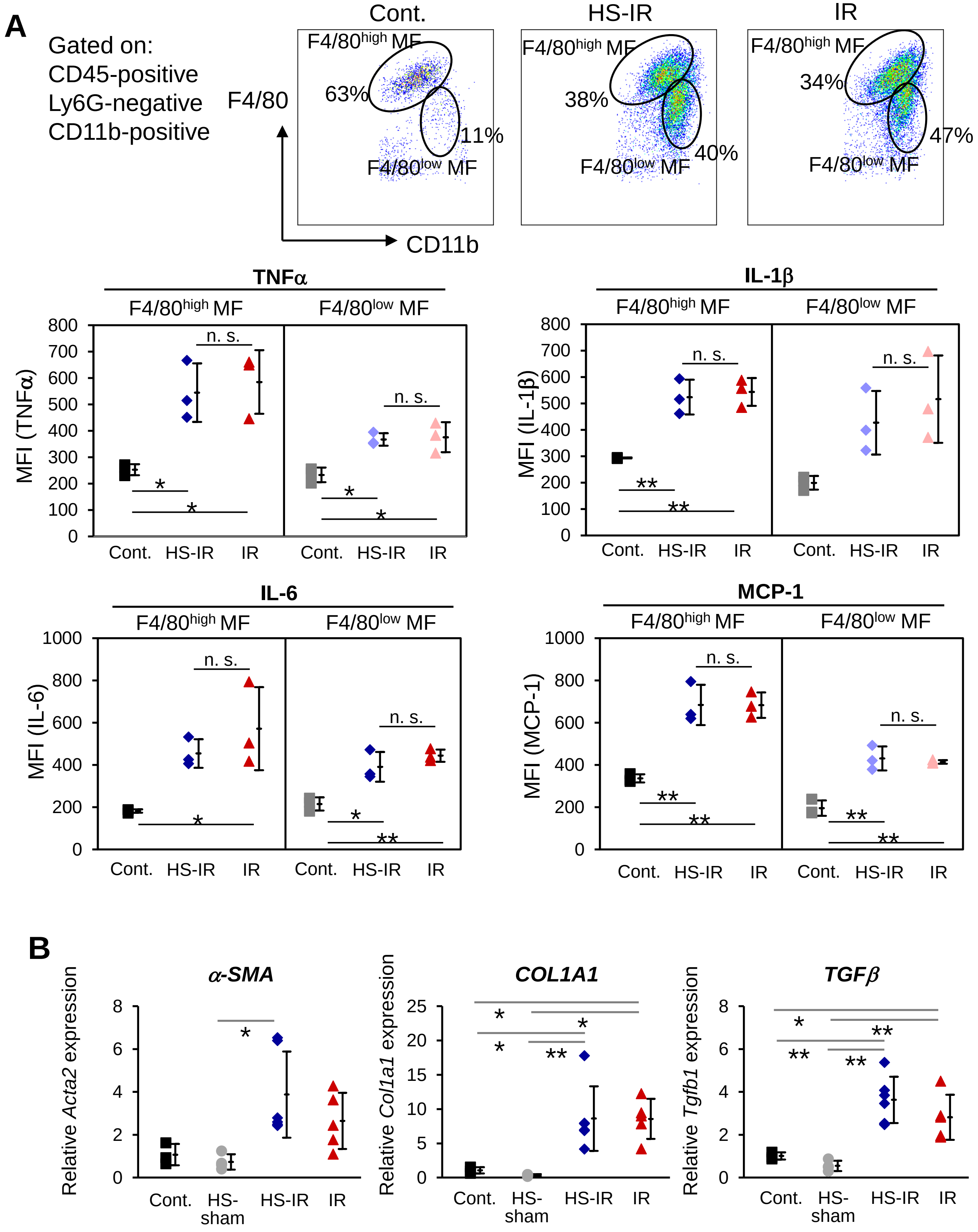

S3 Fig. Changes in pro-inflammatory and fibrotic factors after HS-IR.

(A) F4/80-high or low cells in $CD45^{+}Ly\text{-}6G^{-}CD11b^{+}$ cells prepared from kidneys (indicated in the upper panels) were analyzed for the intracellular expression of various pro-inflammatory factors in HS-IR and IR (*n* = 3 each) mice on day 3 after IR/sham. The results from the mice without any treatment (Cont.) are also presented (*n* = 3). Statistics: one-way ANOVA followed by Holm's *post hoc* test. **(B)** QPCR analysis of the mRNA expression of fibrotic genes performed as in Fig 2H.
