# Supplementary material for: High salt exacerbates acute kidney injury by disturbing the activation of CD5L/apoptosis inhibitor of macrophage (AIM) protein
Source: PLoS One. 2021 Nov 29;16(11):e0260449. doi: 10.1371/journal.pone.0260449 (PMC8629239; doi:10.1371/journal.pone.0260449)
Supplement: S4 Fig — Full-length images of immunoblots for serum IgM-free AIM presented in Fig 3A are shown. Immunoblotting for serum AIM together with recombinant AIM (rAIM) in non-reducing was performed using rabbit anti-mouse AIM polyclonal antibody (rab2, made in-house). After IR, IgM-free AIM at 37 kDa (surrounded by red squares) was increased in the IR group (n = 8) especially on day1 (D1) while no or little IgM-free AIM was detected in the “Pre” condition. In contrast, in the HS-loaded condition (HS-IR group, n = 8), the levels of IgM-free AIM after IR were lower than those in the IR group. IgM-bound AIM and backgrounds are surrounded by blue and green squares, respectively. The serum from AIM KO mice are also presented as negative controls besides HS-IR#6, 8 and IR#7, 8. Calculated IgM-free AIM concentration (μg/mL) is presented under each band. (PDF) [file pone.0260449.s004.pdf]

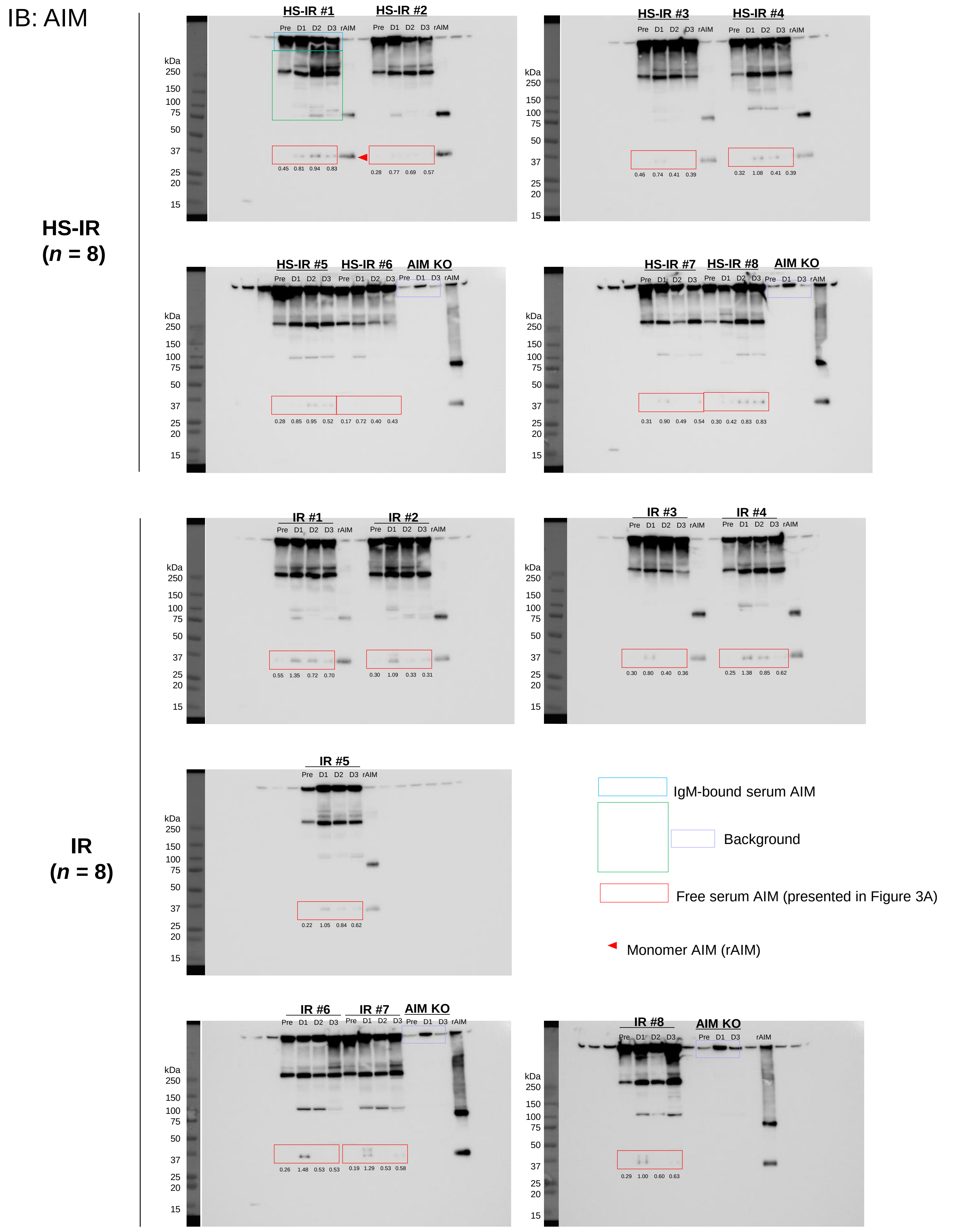
IB: AIM
HS-IR
(*n* = 8)
HS-IR #1
HS-IR #2
Pre D1 D2 D3 rAIM
Pre D1 D2 D3 rAIM
kDa
250
150
100
75
50
37
25
20
15
0.45 0.81 0.94 0.83
0.28 0.77 0.69 0.57
HS-IR #3
HS-IR #4
0.46 0.74 0.41 0.39
0.32 1.08 0.41 0.39
HS-IR #5
HS-IR #6
AIM KO
0.28 0.85 0.95 0.52 0.17 0.72 0.40 0.43
HS-IR #7
HS-IR #8
AIM KO
0.31 0.90 0.49 0.54 0.30 0.42 0.83 0.83
IR
(*n* = 8)
IR #1
IR #2
0.55 1.35 0.72 0.70
0.30 1.09 0.33 0.31
IR #3
IR #4
0.30 0.80 0.40 0.36
0.25 1.38 0.85 0.62
IR #5
0.22 1.05 0.84 0.62
IR #6
IR #7
AIM KO
0.26 1.48 0.53 0.53
0.19 1.29 0.53 0.58
IR #8
AIM KO
0.29 1.00 0.60 0.63
IgM-bound serum AIM
Background
Free serum AIM (presented in Figure 3A)
Monomer AIM (rAIM)

S4 Fig. Full-length images of immunoblots for serum IgM-free AIM

Full-length images of immunoblots for serum IgM-free AIM presented in Figure 3A are shown. Immunoblotting for serum AIM together with recombinant AIM (rAIM) in non-reducing was performed using rabbit anti-mouse AIM polyclonal antibody (rab2, made in-house). After IR, IgM-free AIM at 37 kDa (surrounded by red squares) was increased in the IR group (*n* = 8) especially on day1 (D1) while no or little IgM-free AIM was detected in the “Pre” condition. In contrast, in the HS-loaded condition (HS-IR group, *n* = 8), the levels of IgM-free AIM after IR were lower than those in the IR group. IgM-bound AIM and backgrounds are surrounded by blue and green squares, respectively. The serum from AIM KO mice are also presented as negative controls besides HS-IR#6, 8 and IR#7, 8. Calculated IgM-free AIM concentration (μg/mL) is presented under each band.
