# Supplementary material for: High salt exacerbates acute kidney injury by disturbing the activation of CD5L/apoptosis inhibitor of macrophage (AIM) protein
Source: PLoS One. 2021 Nov 29;16(11):e0260449. doi: 10.1371/journal.pone.0260449 (PMC8629239; doi:10.1371/journal.pone.0260449)
Supplement: S1 Table — (PDF) [file pone.0260449.s005.pdf]

S1 Table. qPCR primer list

Name	Sequence (5'-3')
f-GAPDH	AGAACATCATCCCTGCATCC
r-GAPDH	CACATTGGGGGTAGGAACAC
f-KIM-1	TCCACACATGTACCAACATCAA
r-KIM-1	GTCACAGTGCCATTCCAGTC
f-NGAL	CCATCTATGAGCTACAAGAGAACAAT
r-NGAL	TCTGATCCAGTAGCGACAGC
f-TNFA	CCCTCACACTCAGATCATCTTCT
r-TNFA	GCTACGACGTGGGCTACAG
f-IL1B	CTGGTGTGTGACGTTCCCATTA
r-IL1B	CCGACAGCACGAGGCTTT
f-IL6	TCTATACCACTTCACAAGTCGGA
r-IL6	GAATTGCCATTGCACAACTCTTT
f-MCP1	CATCCACGTGTTGGCTCA
r-MCP1	GATCATCTTGCTGGTGAATGAGT
f-F4/80	CCTGGACGAATCCTGTGAAG
r-F4/80	GGTGGGACCACAGAGAGTTG
f-Acta2	CTCTCTTCCAGCCATCTTTCAT
r-Acta2	TATAGGTGGTTTCGTGGATGC
f-Col1a1	CATGTTCAGCTTTGTGGACCT
r-Col1a1	GCAGCTGACTTCAGGGATGT
f-TGFbeta1	TGGAGCAACATGTGGAACTC
r-TGFbeta1	CAGCAGCCGGTTACCAAG
