# Supplementary material for: High salt exacerbates acute kidney injury by disturbing the activation of CD5L/apoptosis inhibitor of macrophage (AIM) protein
Source: PLoS One. 2021 Nov 29;16(11):e0260449. doi: 10.1371/journal.pone.0260449 (PMC8629239; doi:10.1371/journal.pone.0260449)
Supplement: Attachment — Submitted filename: Responses to Reviewers comments PONE-D-21-26872 211012.docx [file pone.0260449.s006.docx]

**Responses to Reviewers’ Comments**

We thank the referees for their insightful and constructive comments during the initial review of our manuscript. Please note that we received single comment from one reviewer. We have revised the manuscript adequately in response to the concerns raised by the Reviewer as follows (the reviewer’s comments are shown in blue).

We hope that our changes well satisfy the Reviewers.

**Comment.** I have one comment that needs your attention. The huge increase in serum chloride and sodium can only be due to extreme hyperosmolar dehydration secondary to the failure of these very ill mice to maintain fluid intake and possibly polyuria. I also think that this very severe dehydration lead to the very high mortality rather than the accumulation of uremic toxins that typically can be tolerated for longer periods. This is quite common in elderly patients with AKI, see [Hyperosmolar dehydration: A predictor of kidney injury and outcome in hopitalized older adults. El-Sharkawy et al. Clin Nutr. 2020;39:2593-9]. This study should be cited. Maintenance of normal serum urea, creatinine and potassium also suggests that renal function, although impaired as suggested by increased IS, was not yet at end-stage kidney disease. Did you measure hematocrit? If so, please include these data. I suspect a marked increase. If not, please measure total protein or albumin in serum. These are also proxy markers of dehydration (although less accurate than hematocrit). If no serum was saved then at least calculate serum osmolality with the standard simple formula for normoglycemic uremic serum (serum osmolality = 2*Na + 2*K +urea). If you measured urine production/24h please provide these data as well. Urine osmolality would also be useful. Please move the serum Na and serum K data (currently Figure S2) to the main body. Finally, adapt the results and discussion to incorporate these insights.

**Response**. Thank you for the reviewer’s comment. In response to the comment, we addressed the serum levels of total protein and albumin in IR mice with or without high-salt, as well as in sham mice with high-salt to test the presence of dehydration in mice fed with high-salt. As demonstrated in the new Fig 2G, the levels of both items were almost comparable on day 1 to day 3 after IR. Hence, it is unlikely that severe dehydration was the major cause of high mortality and hyperchloremia observed in IR mice fed with high-salt. We described this observation and conclusion in page 16 line 15, also citing the indicated paper (reference 43). In addition, as the reviewer suggested, we have moved the S2 Fig to the main body as Fig 2E and 2F.
